# Genetic polymorphisms of DNA double strand break gene Ku70 and gastric cancer in Taiwan

**DOI:** 10.1186/1471-2407-11-174

**Published:** 2011-05-17

**Authors:** Mei-Due Yang, Hwei-Chung Wang, Wen-Shin Chang, Chia-Wen Tsai, Da-Tian Bau

**Affiliations:** 1 Department of General Surgery, China Medical University Hospital, Taichung, Taiwan, R.O.C; 2 Terry Fox Cancer Research Lab, China Medical University Hospital, Taichung, Taiwan, R.O.C; 3 Graduate Institute of Basic Medical Science, China Medical University, Taichung, Taiwan, R.O.C; 4 Institute of Clinical Medical Science, China Medical University, Taichung, Taiwan, R.O.C

**Keywords:** Ku70, Polymorphism, Gastric cancer, Carcinogenesis

## Abstract

**Background and aim:**

The DNA repair gene *Ku70*, an important member of non-homologous end-joining repair system, is thought to play an important role in the repairing of DNA double strand breaks. It is known that defects in double strand break repair capacity can lead to irreversible genomic instability. However, the polymorphic variants of *Ku70*, have never been reported about their association with gastric cancer susceptibility.

**Methods:**

In this hospital-based case-control study, the associations of *Ku70 *promoter T-991C (rs5751129), promoter G-57C (rs2267437), promoter A-31G (rs132770), and intron 3 (rs132774) polymorphisms with gastric cancer risk in a Taiwanese population were investigated. In total, 136 patients with gastric cancer and 560 age- and gender-matched healthy controls recruited from the China Medical Hospital in Taiwan were genotyped.

**Results:**

As for *Ku70 *promoter T-991C, the ORs after adjusted by age and gender of the people carrying TC and CC genotypes were 2.41 (95% CI = 1.53-3.88) and 3.21 (95% CI = 0.96-9.41) respectively, compared to those carrying TT wild-type genotype. The *P *for trend was significant (*P *< 0.0001). In the dominant model (TC plus CC versus TT), the association between *Ku70 *promoter T-991C polymorphism and the risk for gastric cancer was also significant (adjusted OR = 2.48, 95% CI = 1.74-3.92). When stratified by age and gender, the association was restricted to those at the age of 55 or elder of age (TC vs TT: adjusted OR = 2.52, 95% CI = 1.37-4.68, *P *= 0.0139) and male (TC vs TT: adjusted OR = 2.58, 95% CI = 1.33-4.47, *P *= 0.0085). As for the other three polymorphisms, there was no difference between both groups in the distributions of their genotype frequencies.

**Conclusion:**

In conclusion, the *Ku70 *promoter T-991C (rs5751129), but not the *Ku70 *promoter C-57G (rs2267437), promoter A-31G (rs132770) or intron 3 (rs132774), is associated with gastric cancer susceptibility. This polymorphism may be a novel useful marker for gastric carcinogenesis.

## Background

Gastric cancer is the fourth most common cancer over the world and affects approximately 900,000 individuals every year [1]. Although the identification of *Helicobacter pylori *has revolutionized the understanding of its epidemiology and pathogenesis, the initiation etiology and genomic contributing factors of gastric cancer are still largely unknown [2]. Human DNA repair mechanisms protect the genome from various insults caused by endogenous and environmental agents [3], and mutations or defects in the DNA repairing system are thought to be essential for tumorigenesis [4,5]. Therefore, it is logical to suspect that genetic variants of DNA repair genes might contribute to gastric cancer pathogenesis.

DNA double strand breaks (DSBs) are repaired by the DNA DSB repair system [6,7], which consists of two subpathways, homologous recombination (HR) and nonhomologous end-joining (NHEJ) [8]. In humans, NHEJ is the predominant repair system. At present, several proteins involved in the NHEJ pathway have been identified; namely, the ligase IV and its associated protein XRCC4, the three components of the DNA dependent protein kinase (DNA-PK) complex, Ku70, Ku80, and the catalytic subunit PKcs [9]. Genetic variation in DNA repair genes has been postulated as an important contributor to the aetiology of gastric cancer [10,11]. However, there is seldom information regarding gastric cancer and DNA repair gene polymorphisms. As for NHEJ, genetic polymorphisms influence DNA repair capacity and confer predisposition to several cancers, including skin [12], breast [13-15], bladder [16,17], and oral cancers [18,19].

In our previous study, we have found that one polymorphism of *XRCC4 *DSB repair gene is associated with gastric cancer susceptibility in Taiwan [20]. In this study, we assumed that the upstream gene *Ku70 *in NHEJ, like *XRCC4*, its polymorphisms may also contribute to gastric carcinogenesis. To test this hypothesis, we determined the genotypic frequency of four polymorphisms of the *Ku70 *gene at promoter T-991C (rs5751129), promoter G-57C (rs2267437), promoter A-31G (rs132770), and intron 3 (rs132774), using a polymerase chain reaction (PCR)-based restriction fragment length polymorphism (RFLP) method. To the best of our knowledge, this is the first study carried out to evaluate the contribution of *Ku70 *polymorphisms in gastric oncology all over the world.

## Methods

### Study population and sample collection

One hundred and thirty six patients diagnosed with gastric cancer were recruited at the outpatient clinics of general surgery between 2005-2008 at the China Medical University Hospital, Taichung, Taiwan, Republic of China. The mean age of the gastric cancer patients and the controls were 51.4 (range = 38 to 79, SD = 9.3) and 52.2 (range = 41 to 77, SD = 7.6) years, respectively. All patients voluntarily participated, completed a self-administered questionnaire and provided peripheral blood samples. Five hundred and sixty non-gastric cancer healthy people as controls were selected by matching for age and gender after initial random sampling from the Health Examination Cohort of the hospital. Our study was approved by the Institutional Review Board of the China Medical University Hospital and written-informed consent was obtained from all participants.

### Genotyping conditions

Genomic DNA was prepared from peripheral blood leucocytes using a QIAamp Blood Mini Kit (Blossom, Taipei, Taiwan). The PCR cycling conditions were: one cycle at 94°C for 5 min; 35 cycles of 94°C for 30 sec, 55°C for 30 sec, and 72°C for 30 sec, and a final extension at 72°C for 10 min. Pairs of PCR primer sequences and restriction enzyme for each DNA product are all listed in Table 1. Then, 10 ≥ of product was loaded into a 3% agarose gel containing ethidium bromide for electrophoresis. Ten percent of the samples both in control and patient groups were analyzed of their genotypes by PCR direct sequencing (Genomics BioSci & Tech Co., Taipei) and the consistency is 100%.

**Table 1:** The primer sequences and restriction fragment length polymorphism conditions for *Ku70 *gene polymorphisms.

Polymorphism (location)	Primers sequences (5' to 3')	Restriction enzyme	SNP sequence	DNA fragment size (bp)
promoter T-991C (rs5751129)	F: AACTCATGGACCCACGGTTGTGA R: CAACTTAAATACAGGAATGTCTTG	*Dpn II*	T C	301 200 + 101
promoter G-57C (rs2267437)	F: AACTCATGGACCCACGGTTGTGA R: CAACTTAAATACAGGAATGTCTTG	*Hae II*	C G	298 195 + 103
promoter A-31G (rs132770)	F: TACAGTCCTGACGTAGAAG R: AAGCGACCAACTTGGACAGA	*Mnl I*	G A	226 146 + 80
intron 3 (rs132774)	F: GTATACTTACTGCATTCTGG R: CATAAGTGCTCAGTACCTAT	*Msc I*	G C	160 114 + 46

*F and R indicate forward and reverse primers, respectively.

### Statistical analyses

To ensure that the controls used were representative of the general population and to exclude the possibility of genotyping error, the deviation of the genotype frequencies of *Ku70 *single nucleotide polymorphisms in the control subjects from those expected under the Hardy-Weinberg equilibrium was assessed using the goodness-of-fit test. Pearson's Chi-square test or Fisher's exact test (when the expected number in any cell was less than five) was used to compare the distribution of the *Ku70 *genotypes between cases and controls. Cancer risk associated with the genotypes was estimated as odds ratio (ORs) and 95% confidence intervals (CIs) using unconditional logistic regression. Data was recognized as significant when the statistical *P-*value was less than 0.05.

## Results

There were no significant differences between both groups in their age, sex, and other cancer prevalence. The frequencies of the genotypes of the *Ku70 *promoter T-991C, promoter C-57G, promoter A-31G, and intron 3 polymorphisms in the gastric cancer and control groups are shown in Table 2. The ORs after adjusted by age and gender of the people carrying TC and CC genotypes were 2.41 (95% CI = 1.53-3.88) and 3.21 (95% CI = 0.96-9.41) respectively, compared to those carrying TT wild-type genotype. The *P *for trend was significant (*P *< 0.0001). In the dominant model (TC plus CC versus TT), the association between *Ku70 *promoter T-991C polymorphism and the risk for gastric cancer was also significant (adjusted OR = 2.48, 95% CI = 1.74-3.92). On the contrary, as for the *Ku70 *promoter C-57G, promoter A-31G, and intron 3 polymorphisms, the distributions of these polymorphisms were in Hardy-Weinberg equilibrium but there was no difference between gastric cancer and control groups in the distribution in the genotype frequency at these SNP sites (Table 2).

**Table 2:** The associations between *Ku70 *polymorphisms and gastric cancer risk

	Case (%)	Control (%)	Adjusted OR^a ^(95% CI)	*P*-value
promoter T-991C (rs5751129)				
TT	106 (77.9)	502 (89.6)	1.00 (ref)	
**TC**	**26 (19.1)**	**52 (9.3)**	**2.41 (1.53-3.88)**	**0.0020**
CC	4 (3.0)	6 (1.1)	3.21 (0.96-9.41)	0.0837
*P *for trend			**2.87 (1.37-5.02)**	**<0.0001**
(TC+CC) vs TT			**2.48 (1.74-3.92)**	**0.0005**
CC vs (TT+TC)			2.67 (0.71-9.69)	0.1114
Promoter G-57C (rs2267437)				
CC	95 (69.9)	383 (68.4)	1.00 (ref)	
CG	37 (27.2)	167 (29.8)	0.91 (0.48-1.38)	0.6722
GG	4 (2.9)	10 (1.8)	1.56 (0.55-4.97)	0.4954
*P *for trend			0.93 (0.53-3.34)	0.6164
(CG+GG) vs CC			0.98 (0.63-1.52)	0.8367
GG vs (CC+CG)			1.52 (0.58-5.01)	0.4917
promoter A-31G (rs132770)				
GG	109 (80.1)	459 (82.0)	1.00 (ref)	
GA	19 (14.0)	73 (13.0)	1.16 (0.71-1.93)	0.7763
AA	8 (5.9)	28 (5.0)	1.25 (0.51-2.68)	0.6642
*P *for trend			1.18 (0.68-2.34)	0.9065
(GA+AA) vs GG			1.14 (0.73-1.76)	0.6227
AA vs (GG+GA)			1.03 (0.37-3.01)	0.6673
intron 3 (rs132774)				
GG	113 (83.1)	459 (82.0)	1.00 (ref)	
GC	23 (16.9)	101 (18.0)	0.91 (0.60-1.73)	0.8040
CC	0 (0.0)	0 (0.0)		

^a ^Adjusted by age and gender; the line with ORs that significantly differ from 1.00 are bolded

We have further evaluated the genetic association between gastric cancer in different age range and gender by performing the stratification analysis of the *Ku70 *promoter T-991C genotypes (Table 3). As for those people under the age of 55, the distribution of the *Ku70 *promoter T-991C genotypes was not significantly different between case and control groups. Interestingly, an increased risk was found in those people at the age of 55 or elder who carried TC genotype, compared with TT genotype (adjusted OR = 2.52, 95% CI = 1.37-4.68, *P *= 0.0139) (Table 3). When data were stratified by gender, the significantly increased risk was found in those male carrying TC genotype, compared with TT genotype (adjusted OR = 2.58, 95% CI = 1.33-4.47, *P *= 0.0085). There was no difference found in female (Table 3).

**Table 3:** Distribution of *Ku70 *promoter T-991C genotypes stratified by age and gender in the gastric cancer and control groups

	Case (%)	Control (%)	Adjusted OR^a ^(95% CI)	*P*-value
**Age**				
< 55				
TT	52 (80.0)	238 (90.8)	1.00 (ref)	
TC	11 (16.9)	22 (8.4)	2.18 (0.97-4.88)	0.0600
CC	2 (3.1)	2 (0.8)	4.31 (0.69-27.33)	0.1551
≥ 55				
TT	54 (76.1)	264 (88.6)	1.00 (ref)	
TC	**15 (21.1)**	**30 (10.1)**	**2.52 (1.37-4.68)**	**0.0139**
CC	2 (2.8)	4 (1.3)	2.61 (0.51-11.62)	0.2776
**Gender**				
Male				
TT	69 (76.7)	336 (89.1)	1.00 (ref)	
TC	**18 (20.0)**	**36 (9.6)**	**2.58 (1.33-4.47)**	**0.0085**
CC	3 (3.3)	5 (1.3)	2.85 (0.71-11.36)	0.1477
Female				
TT	37 (80.4)	166 (90.7)	1.00 (ref)	
TC	8 (17.4)	16 (8.7)	2.13 (0.79-6.03)	0.1019
CC	1 (2.2)	1 (0.6)	4.07 (0.31-69.45)	0.3371

^a ^Adjusted by age and gender when appropriate; the line with ORs that significantly differ from 1.00 are bolded

## Discussion

The present study is the first one to investigate the role of *Ku70 *gene polymorphisms, which has never been reported to be associated with gastric cancer risk. Our study revealed that the *Ku70 *promoter T-991C polymorphism was associated with the susceptibility to gastric cancer, but the C-57G, A-31G or intron 3 polymorphisms were not (Table 2), especially in those at the age of 55 or elder, and in male (Table 3). In 2003, Fu *et al *have reported that, *Ku70 *rs2267437 polymorphism (which was called C-61G in their paper) was associated with breast cancer risk [21]. It is reasonable that we did not find a significant association of this SNP with gastric cancer as they did in this SNP with breast cancer in Taiwan population. Instead, we found that the genotype of *Ku70 *rs2267437 was associated with gastric cacner. Since *Ku70 *rs2267437 located in the promoter region, its different genotypes may determine the differential expression level of Ku70 protein in the gastric tissue. Thus, the genotypes of *Ku70 *promoter *T-991C *(rs5751129) may determine the expression levels of Ku70 in different stages, regulate the DNA repair capacity and overall genome stability, and play a role in gastric carcinogenesis. Supporting the idea above, Hu *et al *reported that the expression of TERT and Ku70 proteins was significantly up-regulated in both precancerous lesion and gastric cancer tissues and was significantly higher in gastric cancer tissues than that in precancerous lesions [22]. In the future, the correlation between *Ku70 *rs5751129 genotype and phenotype still needs further investigation. In addition to be associated with oral cancer [18], *Ku70 *rs5751129 polymorphism was also found to be associated with two-side pterygium pathology, which are caused by an uncontrolled cell proliferation like that of a tumor [23].

We have taken into consideration and tried our best to conquer the limitations of this study. First, for the possibility that some findings of further sub-grouping are attributed to chance because of the limited numbers of patients, we just provided the main finding with these 136 gastric patients and 560 (more than four-fold of the number of the patients) non-cancer controls, which is very convincing and reliable with multiple-checked significances (*P *< 0.05 both in genotype and allelic frequencies, and those obvious odds ratios and 95% CIs). Second, the false positive findings are statistically explained by hidden population stratification. But in this study, it seems unlikely that population stratification is a major concern, for all the cases and the controls recruited in this study were drawn from the same Taiwanese ethnic group and the Taiwanese population has relatively homogenous genetic background [24]. Therefore, the potential confounding effect of population stratification for genotyping data is not a major concern. Third, the possible selection bias was taken into consideration and reduced to a lowest level by frequency matching on age and gender between the cases and controls. Forth, from another angle, the frequencies of *Ku70 *polymorphisms variant alleles were similar to those reported in the NCBI website in the Asian population studies, for example C allele frequencies of *Ku70 *promoter T-991C are 5.7% in our control group and 4.2~8.9% for Asian populations in NCBI, which suggest no selection bias for the subject's enrolments in terms of genotypes. Therefore, the need for the present results of *Ku70 *promoter T-991C to be verified in further larger gastric patient population is not so urgent. Of course, further studies in larger population and other functional repair assays should be performed in the future for further findings, particularly those among subgroups by clinical and environmental factors, and clarify the mechanisms involved.

In this study, the genotype distribution of the C allele at *Ku70 *promoter T-991C was significantly higher in the gastric cancer group (12.5%) than in the control group (5.7%). It was also found that patients carrying heterozygous TC for *Ku70 *promoter T-991C had a 2.41-fold higher risk of gastric cancer (Table 2). Combination of heterozygous and homozygous (TC or CC) was almost at the same level (OR = 2.48) for gastric cancer risk (Table 2). All these data suggested that the C allele at *Ku70 *promoter T-991C was a novel and important biomarker for gastric carcinogenesis, and as long as -991C was detected, the carriers were more susceptible to gastric cancer. As for the *Ku70 *promoter C-57G, A-31G or intron 3 polymorphisms, our results indicated that their genetic differences were not associated with gastric cancer risk in Taiwanese population (Table 2).

## Conclusions

In conclusion, this is the first report to investigate the association between *Ku70 *gene polymorphisms and gastric cancer. Our findings suggested that *Ku70 *promoter T-991C, but not Ku70 promoter C-57G, promoter A-31G or intron 3 polymorphisms, was associated with higher susceptibility to gastric cancer. The *Ku70 *promoter T-991C polymorphism might become a potential biomarker for the gastric oncology prediction and this paper may also provide a valuable insight into the gastric carcinogenesis.

## Abbreviations

(DSBs): Double strand breaks; (HR): homologous recombination; (NHEJ): nonhomologous end-joining; (PCR): polymerase chain reaction; (RFLP): restriction fragment length polymorphism; (ORs): odds ratio; (CIs): confidence intervals.

## Competing interests

The authors declare that they have no competing interests.

## Authors' contributions

MDY participated in the collection of the samples and clinical data. HCW participated in the literature reviewing. WSC carried out the PCR-RFLP and sequencing genotyping. CWT performed the statistical analysis. DTB designed the study and coordinated the whole team work. All authors read and approved the final manuscript.

## Pre-publication history

The pre-publication history for this paper can be accessed here:

http://www.biomedcentral.com/1471-2407/11/174/prepub
